# A case of ectopic hamartomatous thymoma: controversy over the designation

**DOI:** 10.1186/s40792-019-0593-x

**Published:** 2019-02-18

**Authors:** Makio Hayama, Seiji Yoshitomi, Maiko Tamura, Nobuhiko Ohnishi, Shigeharu Moriyama

**Affiliations:** 1 Department of Surgery, Japanese Red Cross Okayama Hospital, 2-1-1 Aoe, Kita-ku, Okayama city, Okayama 700-8607 Japan; 2 Department of Pathology, Japanese Red Cross Okayama Hospital, 2-1-1 Aoe, Kita-ku, Okayama city, Okayama 700-8607 Japan

**Keywords:** Ectopic hamartomatous thymoma, Cervical tumor, Subcutaneous tumor, Thymic anlage

## Abstract

**Background:**

Ectopic hamartomatous thymoma, which usually occurs in the lower neck, is a rare benign tumor containing spindle cells, epithelial nests, and adipose tissue. Although the origin of this tumor is still unknown, recent reports suggest that the designation of this tumor is inappropriate.

**Case presentation:**

A 38-year-old with an anterior cervical mass in the suprasternal region of her neck was referred to our hospital. An ultrasound examination showed that the well-defined oval mass was 31 × 23 × 17 mm in size. A non-enhanced computed tomography scan of the neck revealed that the distinct neck mass in the subcutaneous tissue had a mixture of soft tissue and fatty components. The cervical tumor was clinically diagnosed to be an unusual lipoma with degeneration. The patient underwent the neck mass extirpation. During the surgery, the cervical mass was well demarcated and did not adhere to the surrounding tissues. The postoperative course was uneventful. The gross pathology report showed that the neck mass measured 3.0 × 2.5 × 2.0 cm. Microscopically, the tumor was composed of spindle cells, epithelial nests, and mature adipose tissue. Immunohistochemical examination revealed that both spindle cells and epithelial nests were positive for cytokeratin AE1/AE3. These histopathological findings were consistent with the features of ectopic hamartomatous thymoma. Over a follow-up period of 30 months, this patient exhibited no evidence of recurrence.

**Conclusions:**

Ectopic hamartomatous thymoma should be considered in the differential diagnosis of subcutaneous tumors in the lower neck, when the CT shows the tumor has the mixed components of fat and soft tissues.

## Background

Ectopic hamartomatous thymoma (EHT) usually occurs in the lower neck, and it is a rare benign tumor containing spindle cells, epithelial nests, and adipose tissue. The first case of EHT was reported by Smith and McClure in 1982 [1], but the tumor was not designated as EHT at the time. Since its first report in 1982, about 80 cases of EHTs have been reported to date in the English literature [2]. We herein report a case of EHT and further discuss the recent controversy regarding the designation of this tumor.

## Case presentation

**Fig. 1:**
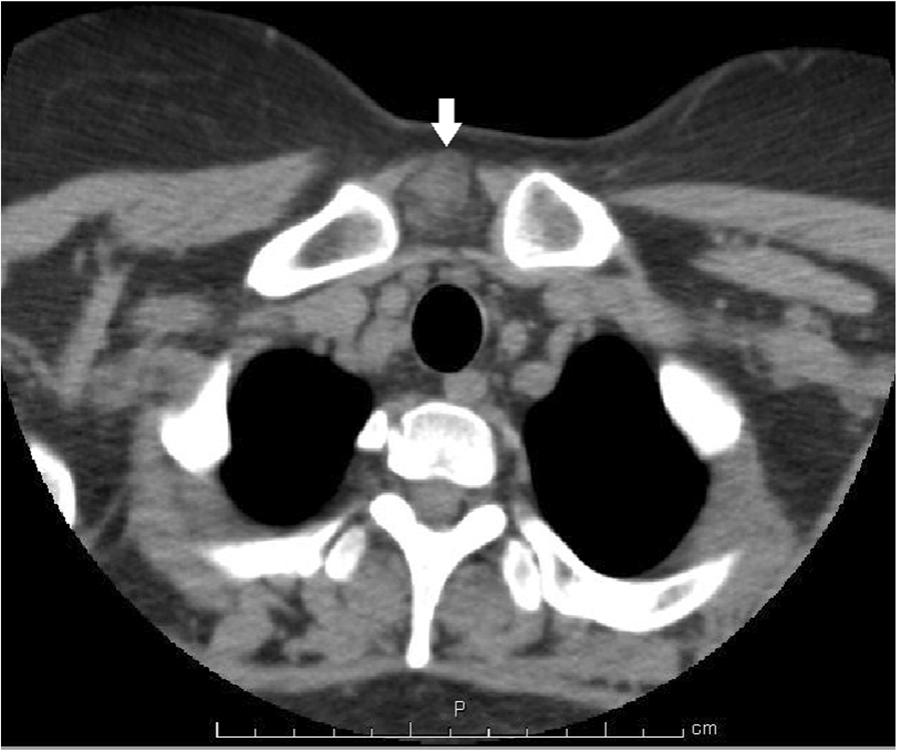
The neck non-enhanced computed tomography scan revealed that the mass in the suprasternal subcutaneous tissue had the mixture components of soft tissue and fat density (arrow)

**Fig. 2:**
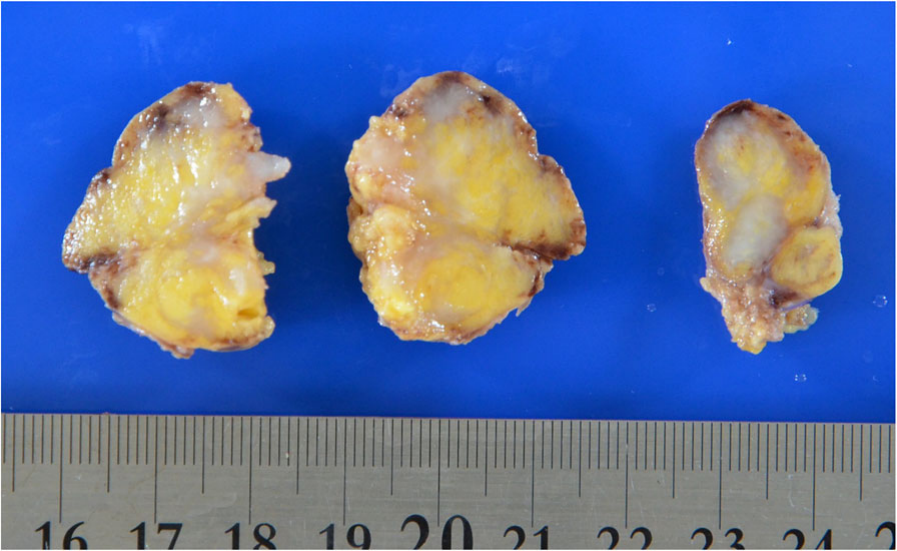
Gross appearance of the resected specimen showed the cut surface of the tumor was whitish, yellowish, and solid

**Fig. 3:**
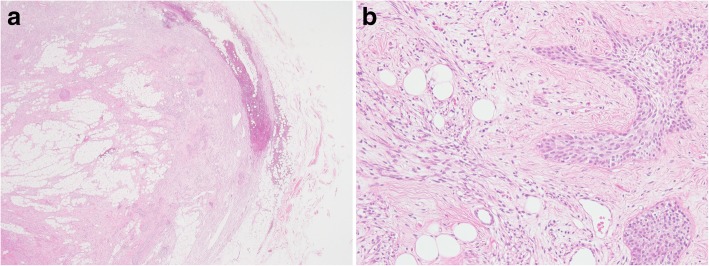
**a**, **b** Histologically, the tumor was composed of an admixture of spindle cells, epithelial nests, and adipose cells (hematoxylin-eosin stain)

**Fig. 4:**
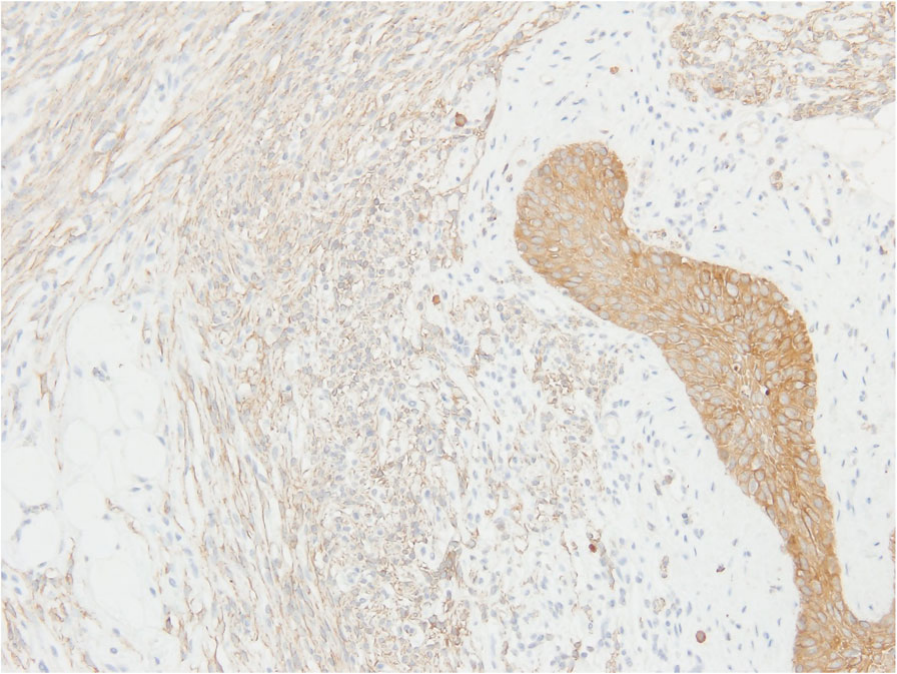
In immunohistochemical examination, the epithelial nests were strongly positive for cytokeratin (CK) AE1/AE3, and the spindle-shaped cells were weakly positive for CK AE1/AE3

A 38-year-old woman with a 3-month history of an anterior cervical mass located in the suprasternal region of her neck was referred to our hospital. Physical examination revealed that the 3-cm movable neck mass was firm and slightly tender on palpation and had a distinct margin from surrounding tissues. An ultrasound examination showed that the well-defined oval mass was 31 × 23 × 17 mm in size and exhibited heterogeneity. Furthermore, a non-enhanced computed tomography (CT) scan of the neck revealed that the distinct neck mass in the subcutaneous tissue had a mixture of soft tissue and fatty components (Fig. 1). Based on these findings, the cervical tumor was clinically diagnosed to be an unusual lipoma with degeneration; however, we could not exclude the possibility of it being a malignant tumor such as liposarcoma. Thus, fine-needle aspiration cytology of the tumor was performed, but no diagnosis of malignant cells was obtained. Further examinations were conducted to address concerns related to her menstrual pain, and abdominal magnetic resonance imaging revealed bilateral ovarian cysts in the lower abdomen. Under general anesthesia, the patient underwent neck mass extirpation and bilateral ovarian cystectomy; pathological examination of the cysts resulted in a diagnosis of ovarian endometriotic cysts. During the surgery, the cervical mass was well demarcated and did not adhere to the surrounding tissues. The postoperative course was uneventful. The gross pathology report showed that the neck mass measured 3.0 × 2.5 × 2.0 cm. The cut surface of the specimen was heterogeneous, solid, whitish, and yellowish (Fig. 2). Microscopically, the tumor was composed of spindle cells, epithelial nests, and mature adipose tissue (Fig. 3a, b). Immunohistochemical examination revealed that both spindle cells and epithelial nests were positive for cytokeratin (CK) AE1/AE3 (Fig. 4). As these histopathological findings were consistent with the features of EHT, a diagnosis of EHT was confirmed. Over a follow-up period of 30 months, this patient exhibited no evidence of recurrence.

## Discussion

Historically, tumors designated as “EHT” were first described by Smith and McClure in 1982 [1]. They reported that EHT is a rare benign neoplasm consisting of fibroblastic, epithelial, and adipose components. In 1984, Rosai et al. [3] named this neoplasm as “ectopic hamartomatous thymoma,” assuming that this tumor was derived from the third branchial arch and composed of thymic tissues. Recently, Sato et al. [2] reviewed 79 cases of EHT in the English literature and demonstrated that EHTs often occur in middle-aged adults (mean age 46 years, range 19–89 years), with a significantly higher ratio in men (male to female ratio, 3.4:1). EHTs are most commonly located in the subcutaneous tissues of the supraclavicular, suprasternal, or sternoclavicular regions [2]. Histologically, EHTs consist of an admixture of spindle cells, epithelial nests, and mature adipose tissues. Although spindle cells seem to originate from mesenchymal tissues, these cells stain positive for CK, highlighting their epithelial features. In terms of spindle cells, synovial sarcoma is difficult to differentiate from EHT [4]. As SYT-SSX fusion genes are found in most synovial sarcoma cases, polymerase chain reaction or fluorescence in situ hybridization is useful to differentiate synovial sarcomas from EHTs in which genetic changes are not detected [4]. In our study, the tumor was composed of spindle-shaped cells, fat cells, and some solid nests of squamous cells. Moreover, immunostaining experiments demonstrated that the spindle-shaped cells were weakly positive for CK AE1/AE3. Based on these histopathological findings, the diagnosis of EHT was confirmed.

Although EHTs are usually benign (including our case), three of 79 EHT cases [2] showed malignant features without any recurrence after complete resection. However, two cases of recurrence where EHTs had been incompletely resected at the time of initial surgery have been reported [5]. In our case, we performed tumor extirpation for pathological confirmation, as we did not reach the final diagnosis preoperatively. Simple resection was sufficient to treat this EHT, although a malignant tumor, such as sarcoma, requires extended resection. Even when a preoperative definitive diagnosis of EHT is obtained, simple but complete tumor resection is recommended.

An accurate nomenclature to describe this rare neoplasm is still under discussion as the exact origin of EHT remains unknown. In 2004, Fetsch et al. [5] proposed a new designation, “branchial anlage mixed tumor,” for EHT based on the following observations in 21 cases: failure to identify any definitive thymic tissue and myoepithelial differentiation of spindle cells. In 2016, Weissferdt et al. [6] proposed a different nomenclature for EHT, i.e., “thymic anlage tumor.” They studied nine cases of EHTs and, based on their findings, reported that the histologic and immunohistochemical features of the EHTs were reminiscent of a thymic derivation, suggesting their possible origin from remnants of the thymic anlage. Another term, “biphenotypic branchioma,” suggested by Sato et al., was supported by their observation that EHTs show dual mesoderm and endoderm derivation [2]. Based on the clinicopathological and immunohistochemical studies relating to EHTs, we agree that use of the term “EHT” should be discouraged as there is no evidence for this tumor being ectopic, hamartomatous, or a thymoma. Despite the similarities between the two diagnostic names, EHT is completely distinct from ectopic cervical thymoma.

## Conclusions

Although this tumor is rare, EHT should be considered in the preoperative diagnosis when CT shows a tumor in the lower neck with mixed components of fat and soft tissues. Finally, more case reports and discussion might be necessary before an appropriate nomenclature of this tumor can be proposed.

## Abbreviations

CK: Cytokeratin
EHT: Ectopic hamartomatous thymoma
